# A Music-Mediated Language Learning Experience: Students’ Awareness of Their Socio-Emotional Skills

**DOI:** 10.3389/fpsyg.2019.02238

**Published:** 2019-10-04

**Authors:** Esther Cores-Bilbao, Analí Fernández-Corbacho, Francisco H. Machancoses, M. C. Fonseca-Mora

**Affiliations:** ^1^Education Department, Faculty of Education, Psychology and Sports Sciences, University of Huelva, Huelva, Spain; ^2^English Studies Department, Faculty of Humanities, University of Huelva, Huelva, Spain; ^3^Methodology and Data Analysis Department, Andalusia Beturia Foundation for Health Research (FABIS), Huelva, Spain

**Keywords:** socio-emotional skills, foreign language learning, music, expressivity, teamwork, mediation

## Abstract

In a society where mobility, globalization and contact with people from other cultures have become its distinctive traits, the enhancement of plurilingualism and intercultural understanding should be of the utmost concern. From a positive psychology perspective, agency is the human capacity to affect other people positively or negatively through one’s actions. This agentic vision can be related to mediation, a concept rooted in socio-cultural learning theory, where social interaction is considered a fundamental cornerstone in the development of cognition. These social interactions in the language learning setting may be facilitated through musical activities due to their social bonding effect. This paper tries to offer insights into how a music-mediated experience in language learning may develop students’ interpersonal and collaborative competences to become active members of a more inclusive society. Mediation, considered to be a paradigm shift in the foreign language classroom and for different out-of-class language learning possibilities, could also provide an environment where learners maximize their emotional intelligence. Our paper focuses on this paradigm shift spearheaded by the Common European Framework for Languages Companion Volume (CEFR/CV) and the considerable repercussions it is bound to have for foreign language didactics, as cooperative tasks become central to foreign language learning. We hypothesize that mediated language learning experiences (MeLLEs) imply a socio-emotional change in learners, focusing on the others, on their needs and interests, by trying to help them understand texts, concepts or facilitating communication with their peers. An intervention with a music-MeLLE was designed and implemented in an L2 classroom of adult learners with divergent backgrounds. A self-assessment scale with mediation descriptors and the socio-emotional expertise scale (SEE) were administered. Results show that students become more mindful of their strengths, and of their capacity for collaboration and teamwork. This leads to more awareness of their mediation skills. Students’ mediation skills correlate significantly with their socio-emotional skills – specifically with their expressivity. The implementation of a music-mediated experience also promoted tolerance and enhanced learners’ intrinsic motivations for language learning at the same time as acknowledging their diversity.

## Introduction: A Socio-Emotionally Enriched Language Education

Language, as a powerful human cultural artifact, mediates our knowledge of the world, our connection to others, and our own thought and self-regulation. All these functions intervene when a person takes the role of mediator – a very much needed helper for those who have difficulties in understanding texts, concepts or in communicating in a foreign language or culture (Piccardo et al., 2019).

In recent years, signs of increasingly narrow-minded attitudes, rejection of those who are different, and even tension between member states (Coste and Cavalli, 2015, p. 6) have been commonplace in European societies. In this context, the notions of mediation and plurilingualism (Piccardo, 2017) have become highly relevant. This concept of mediation, based mainly on Vygotsky (1978), was already included in the *Common European Framework for Languages* (CEFR) published in 2002, but it has gained a more central position within the *CEFR Companion Volume* (CERF/CV) (Council of Europe, 2018), released recently. Piccardo (2017) states that this work on mediation implies a paradigm shift in the foreign language classroom, while Coste and Cavalli (2015) connect it to the urge for educational tools for processes that build up pluricultural and plurilingual competences for collaborative dialogue, leading, in turn, to the development of the acceptance of others and the removal of social inequalities.

North and Piccardo (2016) affirm that “Mediation involves the use of language in creating the space and conditions for communication and/or learning, in constructing and co-constructing new meaning, and/or in facilitating understanding by simplifying, elaborating, illustrating or otherwise adapting the original” (p. 87). Thus, the learning process transcends the individual sphere and enters the interactive space. As explained by Piccardo in the CEFR/CV launch conference (2018), mediation implies a dynamic process of meaning-making through “languaging” (Swain, 2006) and “plurilanguaging” (Lüdi, 2015; Piccardo, 2018), while creating a shared safe “third space” (Kramsch, 2002). In fact, the Council of Europe emphasizes the mediating function of schools, because educating citizens who competently act as mediators relies also on the responsibility of institutions. Mediation fosters a wide range of discourse competences relevant for language learners because they are required to rephrase, to alternate languages, and even to combine and switch genres or oral and written expressions. Thus, language resources are developed through interaction (Coste and Cavalli, 2015, pp. 62–63).

However, a configuration of action-oriented learning experiences that may foster learners’ and that may support teachers’ awareness and predisposition toward the benefits of mediation is still needed. This is the focus of this paper: to explore how a music-mediated language learning experience (MeLLE) may affect students’ motivation, students’ socio-emotional development, students’ foreign language competence, and students’ willingness to cooperate with others. We believe that mediation as a paradigm shift in foreign language learning reinforced through musical activities provides an environment where learners maximize their socio-emotional abilities and their communicative skills.

### Mediated-Foreign Language Learning Experiences

MacIntyre et al. (2016, p. 1) explain humans’ capacity to affect other people positively or negatively through their actions. This capacity, called *agency*, is central in communication and relates to the concept of mediation. Agency means that all of those involved in the teaching and learning process – teachers, students, parents and school staff – can affect one another.

According to Underhill (1999), the good language teacher in his/her role of facilitator knows “subject, methods and internal processes” (p. 144) that “generate a psychological climate that is conducive to high quality learning” (p. 147). Feuerstein and Feuerstein (1991) explain this role of facilitator through their Theory of Mediation which states that adults, parents, or teachers mediate when they prepare the content and select those stimuli that help their learners to achieve success. The term “mediated learning experiences” was originally developed by the Israeli psychologist Feuerstein who worked with traumatized youths after the Holocaust. Oon Seng (2003) describes his work and affirms “When others were modifying materials for those with learning disabilities, Feuerstein chose to invest his energies in modifying those learners directly” (p. 54).

Similarly, the Council of Europe’s preparatory study for the development of the CEFR/CV equates “pedagogic mediation” with successful teaching approaches, which encompass facilitating access to knowledge, collaboratively co-constructing meaning as a member of a group in a learning setting, and generating the necessary conditions by creating, organizing and controlling space for creativity (North and Piccardo, 2016; Piccardo, 2017). Thus, the following actions characterize pedagogic mediation: awakening students’ awareness of the meaning and relevance of the task, ensuring that learners understand the purpose of the task, and monitoring to ensure that pedagogic intentions are shared in order to confirm that learners clearly know what the teacher is asking for (Williams and Burden, 1997). In conclusion, teachers as facilitators and mediators foster students’ desire to participate in the language classroom and promote student autonomy through the teaching of meaningful techniques and strategies that encourage reflection, creativity and agency (Jiménez Raya, 2017).

However, despite the fact that students may also act as mediators, the dynamic relationship among students of the same group is often forgotten, thus overlooking the potential that “any cognitive and affective learning can be substantially enhanced by adroit use of interpersonal and group dynamics” (Ehrman and Dornyei, 1998, p. 2).

Brown (2002) endorses Feuerstein’s mediated learning experiences as an approach that may spur students’ predispositions toward language learning. Socio-cultural theory defines the socialization effect of language (Lantolf et al., 2015), where learning a language goes beyond the acquisition of isolated words to name objects or actions. In our study, MeLLEs – which take an action-oriented approach – learners are regarded as social agents who co-construct meaning while mobilizing their general plurilingual and pluricultural competences. In the words of the CEFR/CV, engaging in mediation activities entails that “…one is less concerned with one’s own needs, ideas or expression than with those of the party or parties for whom one is mediating. One needs to have a well-oriented emotional intelligence, […] empathy for the viewpoints and emotional states of other participants in the communicative situation.” Particularly with regard to cross-linguistic mediation, “[…] this inevitably also involves social and cultural competence as well as plurilingual competence” (Council of Europe, 2018).

Naturally, some individuals are more socially oriented than others (McBrien et al., 2018); thus, encouraging the full development of affective factors such as empathy, respect, tolerance, leadership and cooperation capacities is pivotal in order to achieve successful language learning through mediation, where the main focus is to use language in real-life situations to collaborate with others to co-construct meanings. McBrien et al. (2018) ascribe the learners’ social aptitude to their emotional and social skills, along with their empathy and interpersonal sensitivity. During the validation phase of their socio-emotional expertise scale (SEE), which was performed through the analysis of high-quality socio-emotional interactions, they also identified adaptability and expressivity as two relevant factors to be considered. The interactive space where social interplay occurs is constantly being reassessed by participants in communicative acts and, thus, students’ cognitive, social and affective abilities may be affected by a mental filter which hinders their performance in tasks that require interaction and teamwork. Social interactions in the language learning setting may be facilitated through musical activities due to the social bonding effect of music, because “language enables articulation of what is within us, whereas music strengthens what is shared between us” (Kraus and Slater, 2015, p. 208). In this sense, several authors maintain that the incorporation into the classroom of musical material can entail a series of benefits in terms of socio-emotional expression by creating a relaxed and safe classroom atmosphere (Fonseca-Mora et al., 2011). Tarr et al. (2014) confirm that a “self-other merging” occurs while music-making, but whether this phenomenon takes place while engaging in passive listening or when viewing music videos together is yet to be determined.

A growing body of research evidence explores the complementarity of musical activities in developing both first language and additional languages, based on the reciprocal relationship between music and language as the “two sides of the human communication coin” (Kraus and Slater, 2015, p. 216). Music has been found to be useful in reinforcing the learning of languages and the student’s autonomy (Kerekes, 2015), while making it a more motivating and socializing learning process. Thus, while semantically precise communication is conducted through language, music has been linked to the enhancement of social cohesion within a group, the development of interpersonal skills and community building, because “the great strength of music lies in its facilitation of social bonding and shared emotion” (Kraus and Slater, 2015, p. 217). In fact, one of the major hurdles to succeeding in learning languages is the occurrence of negative emotions when engaging in collaborative activities and when adapting to the classroom environment. Fear, insecurity and shyness, among others, limit or prevent interaction with the group in the learning environment, lessening its pedagogical value. In this sense, emotional arousal induced by familiar musical stimuli (van den Bosch et al., 2013) could counterbalance the affective filters which constrain effective classroom communication. Fonseca-Mora and Herrero Machancoses (2016) assert that the use of music can be the tool that elicits fundamental positive emotions in the classroom: “Melodies and rhythm can create an attractive and enjoyable environment fostering learners’ willingness to participate in the language classroom, similar to the effects on human well-being of music and songs in everyday life” (p. 362).

Despite the potential of music to boost cognitive, affective and social faculties, empirical evidence which fully defines the benefits that musical experiences furnish to adults’ foreign language classroom dynamics is very scarce (Milovanov and Tervaniemi, 2011; Fonseca-Mora and González Davies, 2019). Because language learning is a multifarious activity which draws on the learner’s cognitive, social and affective competences, we hypothesize that music could take on a role as mediator, enhancing motivation and positive emotions, which in turn stimulate the deepening and widening of language learning skills.

### Aims

The purpose of this study is to determine the effects of a classroom-based educational intervention conducted over a 4-week period, designed to mobilize textual and communication-based mediation strategies as well as to further the development of socio-emotional finesse by adult language learners, thus expanding their affective skills and bolstering the relations with their peers within the classroom setting. In order to do so, a music-MeLLE designed to exploit adult language learners’ mediation skills was conceived, placing students in plurilingual classes in the central role of mediators.

Although the role of the teacher as a facilitator of the MeLLE is pivotal and merits further consideration, the scope of the present study is restricted to exploring how this experience influences learners’ mediation competences and socio-emotional expertise.

### Research Questions

1 Does a MeLLE affect learners’ perceptions of their socio-emotional and mediation abilities?
2 Do learners’ perceptions of their socio-emotional expertise and mediation competences differ according to their proficiency level?
3 Are there differences in the outcome of the intervention based on the number of foreign languages spoken by learners?
4 Does the student’s socio-emotional profile relate to their mediation competences after intervention?
5 How do learners value the music-MeLLE?

## Materials and Methods

### Participants

The participants were adult students who were enrolled in an intensive English language course during the 2018–2019 academic year at a language school.

In order to measure the effects of the intervention, a pre–post approach was adopted for a sample of 44 students (65.9% female, 34.1% male) of different nationalities: 36 Spaniards (81.8%) and 18.2% (*n* = 8) from other countries (3 Latvian, 2 Turkish, 1 French, 1 Mexican, and 1 Slovak). Their level of competence in English fluctuated from A2 to C1 levels. Most of them had an A2 level (40.9%, *n* = 18); 14 students belonged to the B1–B2 level group (31.8%), while the remaining 27.3% (*n* = 12) had a C1 level. Research participants were multilingual: 43.2% (*n* = 19) spoke two languages, 31.8% (*n* = 14) three, and 25% (*n* = 11) more than three.

### Instruments and Data Collection

#### Socio-Emotional Expertise Scale (McBrien et al., 2018)

The concept of SEE encapsulates an array of specific cognitive abilities which are relevant to successfully navigating social environments, among which the timing and synchrony of behaviors that support overall social-emotional ability are paramount. The descriptors which comprise the SEE are designed to determine the prevalence of two factors – adaptability and expressivity – in the respondents’ psychological and societal repertoires. The descriptors contributing to each factor construe the students’ ability to adequately engage in social interactions and their ability to competently convey affect and ideas to their peers, respectively. Following McBrien et al. (2018), the items pertaining to “adaptability” appraise the respondent’s ability to adjust to a variety of social and emotional interpersonal situations; the construct of “expressivity,” on the other hand, reflects the individual’s ability to convey emotion to others.

#### CEFR/CV Mediation Descriptor Scales (Council of Europe, 2018)

A2–B2 level-differentiated self-assessment scales with mediation descriptors were developed for collecting data regarding the students’ mediation skills and strategies before and after the classroom experience. Relevant descriptors from the CEFR/CV Mediation Descriptor Scales (MDS) were selected and customized to conform to the research aims of the intervention and to the linguistic competence of the participants. In the analysis, the descriptors were divided into two categories: textual mediation and communication-based mediation. For the purposes of this study, textual mediation focuses on learners’ ability to make the information contained in oral or written texts accessible to others, while communication-based descriptors measure skills mobilized in order to avoid misunderstandings when communication exchanges take place and interaction with others is required.

### Procedure

**FIGURE 1:**
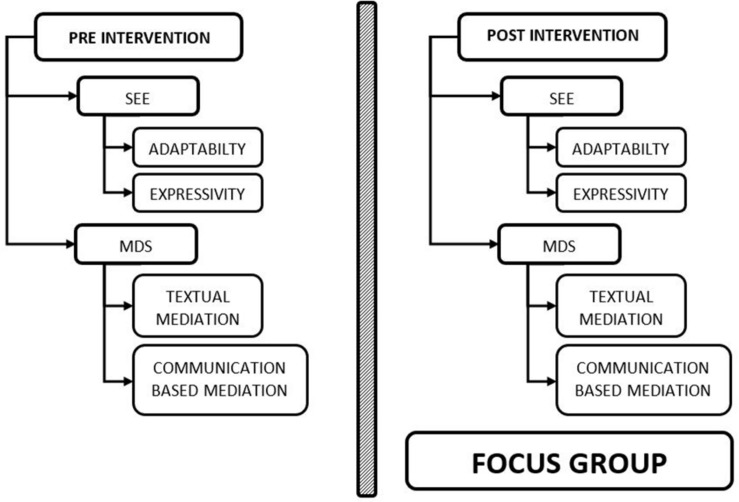
Flowchart of the research design.

First, a music-MeLLE with action-oriented tasks entitled “Plurilingual Songs for Language Learners” was designed. The tasks included require textual and communication-based mediation. Second, the SEE test and the MDS were administered (Figure 1). Permission from principals to carry out the project was requested, and the teachers and students were informed beforehand of the project so that they could express their willingness to participate.

The initial data collection took place in the middle of the second trimester and it was completed again approximately 4 weeks later, once the participants had completed the allotted activities. In order to achieve the set aims, a batch of tasks encompassing music preference and perception and understanding of emotions were proposed. After being informed about the purpose of the study and the tasks they had to complete, the students were asked to cooperatively conduct an analysis of popular music videos of their choice, examining both the visual cues and the lyrics of the songs, and to infer the figurative meaning of the textual elements.

In session one, a modern music video was selected for its visual narrative script and its potential to trigger discussion with the purpose of modeling the activity. A description of its audio-visual and textual elements was elicited from the session. Subsequently, the students were organized into working groups to which different songs were assigned. They were asked to replicate the analysis, focusing on words or expressions connected to feelings and figurative language. As an independent study task, the students looked for examples of songs whose lyrics depict different emotional states; they subsequently brought their lyrics to the next session.

In session two, using the lyrics contributed by the team members, the groups collected examples of vocabulary, idiomatic expressions and metaphors used to articulate feelings. In a whole-class discussion, each group shared and compared their findings with their classmates. Recurrent themes and stereotypes across the examined songs were identified. Back in their working groups, the students were asked to list the features that quality songs should have.

In session three, the students reflected on the importance of deciphering the messages which are implicitly conveyed in popular music videos. Subsequently, they considered the music media consumption choices of youngsters nowadays and the impact that the exposure to certain topics might have on young people’s development, as well as what alternative messages they would like to direct toward adolescents today. The groups were encouraged to discuss how they could use the typical features of songs and music videos to get educational messages across.

As a culminating task, the students were requested to post a blog entry recommending songs which carry positive messages or songs which can help to develop the linguistic competence of foreign language learners. Messages that students produced nominating the best songs for learning foreign languages were required to identify the elements which made those songs stand out for such a purpose. A model for a blog post was provided and comments were included which identified parts, style and features; time was allotted for the students to work on a draft of the text they intended to post online.

Once their texts were complete, they went through a peer editing process and received feedback based on a peer-assessment checklist provided by the teacher. Special emphasis was placed on the register and appropriateness of the reported observation messages, thus honing the affect-related processes that underlie desirable social behavior. When the production of the texts was complete, the students shared their posts on a blog administered by the teacher and were encouraged to post comments about their fellow classmates’ entries.

Students were also asked to fill in the SEE and MDS in view of their music-MeLLE. Finally, a sample of twelve students was re-contacted to partake in a focus group interview, in order to value the mediated experience and supplement the information obtained during the previous quantitative research phase.

### Data Analysis

A mixed method was used to analyze all data obtained.

The statistical analysis includes a univariate descriptive analysis of target study variables to inform the description of the variables in the sample. Non-parametric tests were used, given the non-normality of the variables under study determined by the Shapiro–Wilk and Kolmogorov–Smirnov tests. To ascertain the possible effect of the linguistic proficiency level of the subjects on the test results, the Kruskal–Wallis *H* test for pre- and post-test variables was carried out. The Mann–Whitney *U* test was used to identify the proficiency level groups among which such a difference existed. For the contrast between pre- and post-test moments, and given the non-normality mentioned above, the non-parametric Wilcoxon test was carried out. Finally, a correlation analysis based on Spearman’s Rho was performed.

This quantitative analysis was completed using a qualitative, thematic analysis which revealed students’ evaluation of the learning experience.

## Results

Between the pre- and post-test periods, the response rate remained invariant. In the post-test, fully completed questionnaires were received from 44 students. Descriptive variables were calculated, disaggregated both globally and by gender, and a pre–post comparison was carried out using the Wilcoxon signed rank test, which rendered significant differences in the pre- and post-scores of overall mediation (*W* = −2.197, *p* = 0.028) and the pre- and post-scores of textual mediation (*W* = −3.341, *p* = 0.001). No significant differences were observed in the pre–post socio-emotional variables or in the pre–post communication-based mediation variables (Table 1).

**TABLE 1:** Target study variables mean scores (SD) and pre–post differences.

	**Total sample**	**Male**	**Female**	**Wilcoxon test**	***p***
Pre-socio-emotional expertise	90.34 (13.03)	90.40 (11.02)	90.31 (14.15)	–0.393	0.695
Post-socio-emotional expertise	92.09 (1.25)	90.93 (10.73)	92.69 (10.14)		
Pre-adaptability score	57.52 (8.98)	57.73 (7.90)	57.41 (9.63)	–0.091	0.928
Post-adaptability score	58.41 (6.26)	57.80 (7.17)	58.72 (5.85)		
Pre-expressivity score	32.82 (4.97)	32.67 (3.98)	32.90 (5.48)	–0.802	0.423
Post-expressivity score	33.68 (4.93)	33.13 (4.56)	33.97 (5.16)		
Overall mediation pre	78.11 (15.30)	74.11 (15.48)	80.25 (15.04)	−2.197^a^	0.028^∗^
Overall mediation post	81.70 (16.45)	79.57 (16.05)	82.88 (16.84)		
Pre-textual mediation	72.67 (19.34)	68.16 (19.04)	75.08 (19.41)	−3.341^a^	0.001^∗∗^
Post-textual mediation	83.17 (15.74)	86.20 (13.33)	81.49 (16.93)		
Pre-communication-based mediation	80.60 (15.64)	76.31 (17.08)	82.98 (14.57)	–0.235	0.814
Post-communication-based mediation	79.63 (18.50)	76.15 (20.86)	81.64 (17.11)		

*^*a*^Based on negative ranks (H_0_: POST < PRE). ^∗^Differences were significant at the 0.05 level (2-tailed). ^∗∗^Differences were significant at the 0.01 level (2-tailed).*

With respect to the comparison of results according to CEFR proficiency levels, there were also significant differences in overall mediation scores (KW = 15.411, *p* < 0.01) and textual mediation (KW = 29.827, *p* < 0.01) in its pre-moment, but there were no significant differences observed which were attributable to proficiency levels in the post-intervention nor in the rest of variables in both moments. For both variables, differences were found between the level of competence A and levels B and C; there were no differences between levels B and C. Regarding the pre–post contrast differentiated for each of the CEFR levels, significant differences were only observed in Level A for textual mediation (*W* = −3.245, *p* < 0.01) (Table 2).

**TABLE 2:** Contrasts according to CEFR proficiency levels.

			**CEFR A^c^**		**CEFR B^c^**		**CEFR C^c^**	
					
		***p***	***Z***	***p***	***Z***	***p***	***Z***	***p***
Pre-adaptability score	0.632^a^	0.729	–0.683	0.495	–1.508	0.132	–1.061	0.288
Post-adaptability score	2.368^a^	0.306						
Pre-expressivity score	3.542^a^	0.170	–1.092	0.275	–0.566	0.571	–0.903	0.366
Post-expressivity score	3.421^a^	0.181						
Pre-socio-emotional expertise	1.487^a^	0.475	–1.090	0.276	–1.365	0.172	–1.020	0.308
Post-socio-emotional expertise	2.682^a^	0.262						
Pre-textual mediation	29.827^a^	0.000^∗∗^	–3.245	0.001^∗∗^	–0.665	0.506	–1.011	0.312
A–B	0.500^b^	0.000^∗∗^						
A–C	4.000^b^	0.000^∗∗^						
B–C	59.000^b^	0.321						
Post-textual mediation	4.988^a^	0.083						
Pre-communication-based mediation	2.617^a^	0.270	–1.336	0.181	–1.379	0.168	–0.059	0.953
Post-communication-based mediation	5.086^a^	0.079						
Overall mediation pre	15.411^a^	0.000^∗∗^	–1.327	0.185	–1.119	0.263	–0.222	0.824
A–B	47.500^b^	0.003^∗∗^						
A–C	21.000^b^	0.000^∗∗^						
B–C	63.500^b^	0.459						
Overall mediation post	5.367^a^	0.068						

*^*a*^Kruskal–Wallis test. ^*b*^Mann–Whitney *U*, not corrected for ties. ^*c*^Wilcoxon signed ranks test. ^∗∗^Differences were significant at the 0.01 level (2-tailed).*

When conducting pre–post intervention contrasts according to the number of languages spoken by the subjects under study, there were significant differences in textual mediation in learners who speak two languages (Zw = −2.788, *p* < 0.01) and in those speaking three languages (Zw = −2.040, *p* = 0.041), with no differences observed in students who speak more than three languages or for the rest of the variables under study (Table 3).

**TABLE 3:** Wilcoxon signed ranks test pre–post based on number of languages spoken.

	**2 Langs**		**3 Langs**		**+3 Langs**	
			
	***Z***	***p***	***Z***	***p***	***Z***	***p***
Pre–post adaptability score	−1.265	0.206	−0.910	0.363	−1.070	0.285
Pre–post expressivity score	−0.262	0.793	−0.315	0.752	−1.607	0.108
Pre–post socio-emotional expertise	−1.148	0.251	−0.816	0.414	−1.376	0.169
Pre–post textual mediation	−2.788	0.005^∗∗^	−2.040	0.041^∗^	−0.153	0.878
Pre–post communication-based mediation	−0.840	0.401	−0.051	0.959	−0.771	0.441
Pre–post overall mediation	−1.155	0.248	−1.570	0.116	−0.890	0.374

*Based on negative ranks (H_0_: POST < PRE). ^∗^Differences were significant at the 0.05 level (2-tailed). ^∗∗^Differences were significant at the 0.01 level (2-tailed).*

Finally, the relational study between the general scores in mediation, and the general and differentiated variables of socio-emotional skills after the intervention revealed a significant relationship between the general mediation and socio-emotional scores (ρ = 0.409, *p* < 0.01). When the general socio-emotional variable was disaggregated into its components of adaptability and expressivity, we only found a significant relationship between the overall mediation score and the expressivity score (ρ = 0.465, *p* < 0.01) (Table 4).

**TABLE 4:** Spearman’s rho correlations between target variables.

		**Overall mediation post**
Post-socio-emotional expertise	ρ	0.409^∗∗^
	*p*	0.007
	*N*	42
Post-adaptability score	ρ	0.246
	*p*	0.116
	*N*	42
Post-expressivity score	ρ	0.465^∗∗^
	*p*	0.002
	*N*	42

*^∗∗^Correlation is significant at the 0.01 level (2-tailed).*

## Qualitative Findings

In order to better understand the perceptions of the participants, a focus group interview was conducted. Twelve students were selected so that language proficiency levels would be equally represented; the Spanish and foreign ratio was balanced. They were invited to talk about their classroom experiences: what they had liked the most and what they least minded about the collaborative approach taken in this intervention, whether they favored further use of musical media to inspire their language learning, and whether they had become aware of anything new about themselves.

A thematic analysis of the students’ responses was made by applying a four-step process to synthesize the data. Two of the authors independently reviewed the focus group transcript and identified recurring themes mentioned by participants. Then, the script was coded and the informative extracts were transferred to an *ad hoc* designed data extraction sheet. Finally, themes were grouped into two categories (Tables 5, 6), subdivided into four key concepts: (1) music as a plurilingual and pluricultural mediator; (2) musical activation of agency and positive emotions; (3) teamwork as a valuable and enjoyable experience; and (4) flexibility and coping with uncertainty.

**TABLE 5:** Students’ views on the music-mediated experience.

**Music as a plurilingual and pluricultural mediator**	
	“…trends in current music videos, fashions, everything we have discussed in class, gender equality, social critique, anti-sexist movements, those topics are present in music now more than ever before.”
	“The chance of finding out how people from other cultures process information is great.”
	“The forms of society and lifestyle are changing; in some countries they develop and change before they do in others and it is through music that young people are coming to learn about those changes.”
	“Music inoculates people with these messages (of cultural change)”
	“I am now also wiser in terms of dealing and understanding Spanish culture’s mindset.”

**Musical activation of agency and positive emotions**	
	“We have been able to describe the different songs that each of us contributed and to examine what they conveyed.”
	“Choosing the song, talking about feelings and so on, and writing the text interpreting the author’s intention was interesting.”
	“We like to find the emotions, what the lyrics want to express. As it is another language, they are expressed differently, the translations cannot be done literally, the language is figurative.”
	“(In class) We worked to recognize the emotions and what they meant to us.”

**TABLE 6:** Students’ views on the progress of their interpersonal dimension.

**Teamwork as a valuable and enjoyable experience**	
	“We learn more together because we exchange knowledge.”
	“When one of us had difficulties in understanding something or was not sure on what to do, we would take the time to explain and help them in order for everyone to be on the same page.”
	“Because we had to speak and explain the matter as clear as possible in a language that was not our mother tongue – this way all my partners and I experienced the importance of speech in language learning and its impact on the different reactions of my classmates.”
	“It was nice to get to work with people that I had never met and with an Erasmus student, as I do not get that opportunity very often.”
	“Working as a team was very enjoyable, mainly because we were very organized and distributed the tasks evenly, everyone in the group knew what they had to do and did it to the best of their abilities.”
	“Working in a team has always been challenging for me, as I love doing things independently. You never know if you can fully count on people, especially on the ones you do not know.”

**Flexibility and coping with uncertainty**	
	“I was dazed, for we had never had classes like this before.”
	“Initially I felt slightly uncomfortable, and also baffled because I didn’t know which song to select.”
	We sometimes have had some different thoughts about how to carry out the project, but at the end we always reached a common agreement.”
	“The problem with English was a barrier as well. Our levels ranged from B1 to C1 and that level gap was a huge problem when explaining our ideas, so we helped each other.”
	“It has been a challenge because we are not used to doing teamwork activities, and I still have a lot to learn.”
	“I enjoyed the discussions because everyone had their own opinion that only meant that we care about the result and it only made the ideas more thought through.”
	“Each group leader should give directions to the members of the group […] Otherwise, the other members of the group would be distracted even by a single person who did not perform what was asked of them. Unfortunately, this is what we experienced.”

Analyzing the audio-visual narratives of music videos produced in different countries led to a better understanding of some traits of the target culture, and some reflections also made evident the awakening of the students’ pluricultural and plurilingual competences. Several students highlighted the opportunity to showcase their work, as well as to access their classmates’ contributions and provide feedback:

“I think the most interesting thing was networking with other communities in other countries. That was the most powerful part. Because, after all, posting something on the Internet is easy, but it doesn’t always allow you to connect with someone who is studying English out there.” (Luis B.)

“Posting our blog entry, seeing our final product online and getting to read what the rest of my classmates have produced, reading everyone’s posts and seeing how our own blog was taking shape was really cool.” (José Ignacio C.)

In general terms, students valued collaborative work as a rewarding and enriching experience. Some of them even openly expressed what they considered to be successful teamwork practices. Conversely, they were displeased when one team member failed to contribute equally to the collective effort; this was the main reason for their reluctance to work with others. On the whole, their reflections show that they were willing to collaborate with others in order to attain the final result. This implies that they managed to join forces to co-construct meaning, to understand new concepts and to access information. That is, they actively assumed the role of mediators. Related to this, students also considered that working with students from other nationalities was an enriching experience which could help them improve their language skills.

A significant number of interviewees reported having felt confused or overwhelmed during the initial stages of implementation of the lesson plan due to a lack of familiarity with the approach. However, they showed awareness of the importance of overcoming uncertainty when interacting with others and of the transferability of the skills acquired to everyday life situations. In their view, striving to come to an agreement in order to achieve a shared goal will be commonplace in the different contexts that they will encounter in their professional careers and personal lives. Consequently, students also felt proud of their ability to overcome the difficulties they had encountered during the learning experience. On the other hand, learners showed concern about difficulties met which had not been easily solved. They also expressed their need to further learn how to handle critical situations, similar to those experienced in teamwork.

Regarding this last issue, on one occasion the students complained to the teachers about the uncooperative attitude of one of their team members. When asked whether they had raised the issue and discussed it, they reported that they did not want to stir up a conflict with that person. It seemed that they were unsure about how to address the problem.

Finally, according to the learners’ self-reported perceptions, music-MeLLEs seem to give prominence to reciprocity and negotiation of goals and outcomes, as set out by the teacher and pursued by the learners. They acknowledged the sense of practicality of this musical MeLLE because it is rooted in reality, in the immediate context of the students. In the adult education context, learners are often notably goal-oriented. The students also acknowledged the integration of diversified activities in its design, which they considered to be adaptable to different levels of complexity and well sequenced, with increasing difficulty to challenge and engage them.

## Discussion and Conclusion

All the different research questions have been addressed by our study. The main aim of our study was to explore whether a music-MeLLE could potentially affect students’ awareness of their socio-emotional and mediation skills while learning foreign languages. The most obvious finding to emerge from the analysis is that the MeLLE had an impact on students’ awareness of their mediation skills, more significantly of their textual mediation skills.

The next question in this study sought to determine the co-dependency of language proficiency level and mediation skills. Prior to the intervention, overall and textual mediation competences were underdeveloped or overlooked by students with lower levels of CEFR proficiency, whereas students with an intermediate or advanced command of English displayed a sound knowledge of their mediation skills and strategies. This seems to indicate that once an intermediate level of proficiency is reached in the foreign language, trainees feel more capable of mediating a text. A possible explanation for these results may be the lack of cross-cultural communication encounters undertaken by students at the initial stages of foreign language acquisition, as compared with a greater exposure to speakers of other languages that learners experience during subsequent years of language learning. However, after the intervention presented in this study, those differences between language proficiency groups were no longer observable, because the learning experience triggered a noteworthy increase in textual mediation scores as well as in overall mediation measures of the least proficient students. This might imply that the MeLLE assisted in making visible the usefulness of pre-existing cultural and language resources and gave the learners a sense of confidence to manage intercommunity relations (Coste and Cavalli, 2015).

As far as the number of foreign languages spoken is concerned, the data obtained shows that students who speak three languages outperformed, in textual mediation activities, their peers who spoke two languages. However, on the question of whether the number of languages spoken by the learners influenced the outcome of the intervention, it seems that the MeLLE benefits most those students who speak only one foreign language. This result may be explained by the fact that those who speak more foreign languages feel that their textual mediation competences have reached their full potential. The knowledge of at least two foreign languages seems to favor the natural development of text mediation skills. Hence, the adoption of a plurilingual take on language education, which focuses on pursuing the learners’ personal development, self-awareness, linguistic and critical awareness, and interculturality (Piccardo et al., 2019) is supported. The results obtained also suggest that similar interventions could probably offset the shortcomings of monolingual and bilingual education programs. Thus, in contexts in which implementing plurilingual teaching approaches is not feasible, interventions based on MeLLE would possibly parallel the students’ perceptions of their mediation skills with those of more linguistically diversified, accomplished students.

However, the most remarkable result derived from the statistical data analyzed is the highly significant correlation between overall mediation competence and socio-emotional expertise. Therefore, our MeLLE affected students’ awareness of their role as agents in interpersonal communication and this awareness linked to learners’ socio-emotional portraits. When examining separately the two contributing factors of learners’ socio-emotional profiles, expressivity and adaptability, it was the former that resulted in a more significant correlation. Because expressivity has been defined as “the ability to successfully convey affect and ideas to other people” (McBrien et al., 2018, p. 1), this could further indicate the benefits of adopting MeLLE in the language classroom.

Additional advantages of MeLLE are highlighted by the qualitative results collected during the focus group interview. The students’ documented perceptions when exposed to the music-MeLLE seem to endorse the suitability of this approach to induce a measurable improvement of their wellbeing and a positive language learning setting, as well as to raise learners’ awareness of their role as social agents within the communicative act. By the end of the intervention program, the students were familiar with the concept of mediation and had got used to working collaboratively. Students showed enthusiasm during teamwork tasks and they were able to show flexibility by overcoming the difficulties encountered. Besides, the students reported that these types of activities made them conscious of their own learning process. They recognized the importance of active listening, respecting everyone’s opinions, and taking on an agentic role by providing feedback to the work of their peers. Moreover, they were satisfied with the common effort to find a shared language and to succeed in the tasks. However, they also demonstrated their need to learn more about how to manage personal accountability for the tasks assigned to each team member. Driven by the above-mentioned benefits, they were willing to engage in MeLLE in the future.

As for the role of music, the qualitative findings obtained in our focus group interviews appear to be consistent with the idea that songs can evoke positive emotions in the foreign language classroom (Fonseca-Mora and Herrero Machancoses, 2016). In this sense, learners highlighted the motivating potential of working with music of their choosing and sieving through familiar lyrics, discovering messages and emotional states expressed therein. These findings seem to correlate favorably with the studies conducted by van den Bosch et al. (2013) and further support the idea that familiarity with self-selected music may amplify the benefits of music-based classroom activities. Similarly, the interviewees reported a renewed feeling of group conscience and sense of belonging brought about by the musical input, which is coherent with the bonding effect mentioned by Tarr et al. (2014) and Kraus and Slater (2015).

The results recorded so far highlight the appropriateness of introducing these types of music-mediated experiences to the language classroom in order to improve students’ development of mediation skills, awareness of their socio-emotional skills and of other people’s perspectives or intentions (Steinbeis and Koelsch, 2009). By developing empathy toward others and social cognition, explaining and predicting other people’s behavior, language learners become more socio-emotionally oriented and prepared to narrow intercultural gaps and social inequalities.

## Limitations and Future Research

The tentative results of this study suggest the need to carry out a broader longitudinal study over an extended period of time to empirically confirm the effects of this type of pedagogic intervention on foreign language adult learners.

## Data Availability Statement

The datasets generated for this study are available on request to the corresponding author.

## Ethics Statement

Ethical review and approval was not required for the study on human participants in accordance with the local legislation and institutional requirements. The patients/participants provided their written informed consent to participate in this study.

## Author Contributions

MF-M: conceptualization, investigation, and funding acquisition. FM: methodology and formal analysis. EC-B, AF-C, FM, and MF-M: writing – original draft. EC-B, AF-C, and MF-M: writing – review and editing.

## Conflict of Interest

The authors declare that the research was conducted in the absence of any commercial or financial relationships that could be construed as a potential conflict of interest.
